# Cell membrane-coated nanoparticles: An emerging antibacterial platform for pathogens of food animals

**DOI:** 10.3389/fvets.2023.1148964

**Published:** 2023-03-06

**Authors:** Sidra Altaf, Khalid Ali Alkheraije

**Affiliations:** ^1^Department of Pharmacy, University of Agriculture, Faisalabad, Pakistan; ^2^Department of Veterinary Medicine, College of Agriculture and Veterinary Medicine, Qassim University, Buraidah, Saudi Arabia

**Keywords:** bacteria, nanoparticles, cell, infection, animals, antibiotic, food

## Abstract

Bacterial pathogens of animals impact food production and human health globally. Food animals act as the major host reservoirs for pathogenic bacteria and thus are highly prone to suffer from several endemic infections such as pneumonia, sepsis, mastitis, and diarrhea, imposing a major health and economical loss. Moreover, the consumption of food products of infected animals is the main route by which human beings are exposed to zoonotic bacteria. Thus, there is excessive and undue administration of antibiotics to fight these virulent causative agents of food-borne illness, leading to emergence of resistant strains. Thus, highprevalence antibiotic-resistant resistant food-borne bacterial infections motivated the researchers to discover new alternative therapeutic strategies to eradicate resistant bacterial strains. One of the successful therapeutic approach for the treatment of animal infections, is the application of cell membrane-coated nanoparticles. Cell membranes of several different types of cells including platelets, red blood cells, neutrophils, cancer cells, and bacteria are being wrapped over the nanoparticles to prepare biocompatible nanoformulations. This diversity of cell membrane selection and together with the possibility of combining with an extensive range of nanoparticles, has opened a new opportunistic window for the development of more potentially effective, safe, and immune evading nanoformulations, as compared to conventionally used bare nanoparticle. This article will elaborately discuss the discovery and development of novel bioinspired cell membrane-coated nanoformulations against several pathogenic bacteria of food animals such as *Klebsiella pneumoniae, Escherichia coli, Staphylococcus aureus, Salmonella enteritidis, Campylobacter jejuni, Helicobacter pylori*, and Group A Streptococcus and Group B Streptococcus.

## Introduction

Livestock and aquatic animals are the major sources of income, food, fertilizer, clothing, and building materials for a human being (1, 2). The continuous progression and growth of livestock sector offer significant opportunities for food security gains, poverty decline, better human nutrition, and agricultural development (3, 4). Farm animals constitute 40% of total agriculture output globally (5). These production animals support the livelihoods and the income of almost 1.3 billion people in the world (6). However, disease outbreak in livestock and aquaculture species may significantly reduce this quantum (7, 8). Highly contagious diseases especially the bacterial diseases of animals may have ripple effects on the revenue, food supply, trade, and even human health as these may lead to reduced animal fertility, low yields (milk, eggs, etc.), animal death, and disease transfer in humans (9). There is a need to better understand the emerging animal diseases and their impact on the environment and society, as well as the development of innovative and potential therapeutic and preventive methodologies for their management.

Several therapeutic approaches have been considered to control livestock and fish bacterial diseases including the use of chemicals and antibiotic drugs (10–16). The use of antibiotics has become a vital input in food animal species to achieve the ultimate goal of successful farming with high production (17–19). Yet, the extensive use of antibiotics has raised the prevalence of antimicrobial resistant bacteria (20). In addition, the existence of drug residues in food animals further exaggerates the alarming situation for human beings (21–23). This lethal condition motivates scientists to search and develop new innovative nano-therapeutic agents which may successfully treat the resistant infections and avoid the undue excessive use of inaffective antibiotic drugs (24, 25). Several groups reported the development of nanoparticles which have been comprehensively analyzed in therapeutic context (26, 27). These tiny particles offer advantages of improved delivery of drug to target site (28) as well as controlled release of cargo (29). However, despite of these benefits, food and drug administration (FDA) approved only a few nanoparticles for clinical use. As our body system can easily recognize and rapidly remove the foreign substances *via* reticuloendothelial system, complex circulatory proteins and immune cells (30). Moreover, another strategy has been considered for surface modification of nanoparticles, that is the coating of Poly(ethylene glycol) (PEG) onto nanoparticles (31). Though pegylation was not a successful solution, as the studies showed that subsequent dosing of PEG-modified nanoparticles induce the rapid clearance by the hepatic phenomenon of accelerated blood clearance (32) *via* IgM antibodies production and complement system activation against PEG (33). Hence, a protective shield for cargo was needed in the form of an optimal delivery system to avoid rapid degradation and to offer a targeted and controlled drug delivery (34). Hence, the idea of development of biomimetic system start gaining attention to functionalize the nanoparticles with therapeutic potential. The cell membrane coating technology involves use of the cell membrane as drug-carrier, facilitating the controlled and targeted delivery of core nanoparticles irrespective to properties of nanomaterial. The cell membrane coated nano-moieties replicate the shape, surface composition, and movement of normal physiological cells (35).

A recent approach is the use of biomimetic nanoparticles in medical science for rapid diagnosis and biocontrol or treatment of bacterial diseases (36–39). Application of biomimetic nanoparticle can provide a sustainable and natural solution to control resistant bacterial infectionsin the livestock and fish farming industry (40, 41). The bioinspired nanotherapy may present a viable antibiotics alternative (40). The cell membrane coatings over nanoparticles give the appearance of surfaces of naturally occurring cells in the body, that when correctly selected and prepared do not have any risk of immune system activation and attack (38). The biomimetic therapeutic design demonstrates several therapeutic benefits like capability of biointerfacing (42, 43), biocompatibility (44), prolonged duration of circulation (45), immune evasion (46), and protection of the entrapped drug from active targeting and degradation (47).

A number of biomimetic nanoformulations have been designed using wide range of natural cell membranes coated over a variety of nanoparticles to ascertain antibacterial potential (48–52). Studies indicated that there is a significant medical importance of biomimetic and bioinspired nanoparticles to fight against the pathogenic bacteria of food animal host (45). However, our understanding of the design strategies for fabricating potentially effective and economically viable antibacterial nano-moieties remains limited. In this review, we will highlight the recent progress on the antibacterial activity of combo of different nanoparticles with natural cell-derived surfaces. Moreover, we will discuss recent research studies regarding successful development of novel biomimetic and biodegradable antibacterial nanomedicines against major bacterial pathogens responsible for veterinary bacterial diseases.

## Concept of cell membrane coating over nanoparticles

The conception of coating several types of nanoparticles with plasma membranes of cells was raised in view of the troubleshooting of the functionalization of synthesized nanoparticles (53). Therefore, scientists developed new nature inspired nano-transporters, so that the nanoparticles may not be degraded by the internal environment of the organism and may easily reach their therapeutic targets (54). The synthesis of biomimetic nanoparticles combines the properties and specifications of cell membranes (external surface) and nanoparticles (core) (55, 56). This new emerging cell membranes coating technology allows the designing of biomimetic nanotransporters with covering surfaces which may directly imitate complicated functionalities of the cells, needed to have precise physiological interaction with other living cells and tissues (57, 58).

The plasma membrane coating concept was first reported in 2011, Hu and his coworkers (59) first employed red blood cell membranes for coating poly (lactic-co-glycolic acid) (PLGA) nanoparticles. The RBCs were first chosen for coating as they have extended circulation period of 120 days so the nanocarriers may stay longer in the blood circulation, the property which is highly needed for the nanocarriers. Since then, for this technology, wide variety of cell types have been considered including mesenchymal stem cells, bacterial cells, platelets, white blood cells, and cancer cells (60, 61).

## Cell membrane coating techniques

To produce cell membrane-coated nanoparticles, the specific cell membrane must be coated over the nanoparticle core (62). The most frequently mentioned technique for cell membrane coating is membrane extrusion and ultra-sound to date (63). Membrane extrusion is one of the primary cell membrane-coating techniques. Physical membrane extrusion is performed so that both the cell membranes and nanoparticles may move through membranes of various pore sizes concurrently, leading to the wrapping of the membranes over the nanoparticles (64). This method is beneficial as uniformly sized particles are obtained by using this methodology (65). The strategies employed for extrusion of membranes are membrane emulsification, vesicle extrusion and precipitation extrusion for emulsions, liposomes and nanoparticles/nanofibers respectively. The extrusion technique was first reported for uniform coating of PLGA nanoparticle core with red blood cell membrane (59). After that successful coating several research groups have stated this method for coating different types of cell membranes with varying pore size (66–68); however, mechanical forces may affect the structure of the membrane in this protocol (59).

Consistent and stable cell membrane-coated nanoparticles (CMCNPs) can be obtained by ultrasound technique. This technique also encourages the membrane and nanoparticles to naturally form a core-shell structure with a little loss of substantial material; however it may destroy the nanoparticles (69). Several researchers have reported to use this technique for assembling different types of cell membranes such as assembling or coating of membranes of platelet (54), stem cell (70), neutrophil (71) onto PLGA nanoparticles; Moreover, coating of cardiac stem cell membrane over microparticles of PLGA (72). Another example is assembling of a hybrid of platelet and RBC membranes over gold nanowires (73).

In another technique, nanoparticles and cell membrane vesicles are first mixed in the microfluidic system with the S and Y-shaped channels. Then, instantaneous pores are created on the vesicles by using electrical pulses at the exit of the channels and thus allowing nanoparticles to penetrate the vesicles, this technique is known as electroporation (74). An example of application of this technique is successful coating of RBC membranes onto ferric oxide (Fe_3_O_4_) nanoparticles resulting in uniform fabrication of RBC-Fe_3_O_4_ nanoparticles of good colloidal stability (75).

Another technique involves co-incubation of nanoparticles and live cells followed by the addition of serum-free media for secretion and production of exosomes (76). While wrapping the cell membrane over nanoparticles, it is vital to keep in mind that the coating process makes stable homogenous nanoparticles that do not interfere with either the function of the nanoparticles or the cellular membrane (77).

Concerning the membrane coating methods, the easiest strategy for coating among the above mentioned methods, is based on subsequent extrusion technique. Moreover, ultrasounds produce sonication forces to coat membrane surfaces over nanoparticles with better efficacy (69). Microfluidic technology is highly recommended for coating magnetic nanoparticles with good control as fine-tuning may be obtained *via* the flow speed (75).

## Bioinspired nanotherapies against principal food borne pathogens

**Figure 1:**
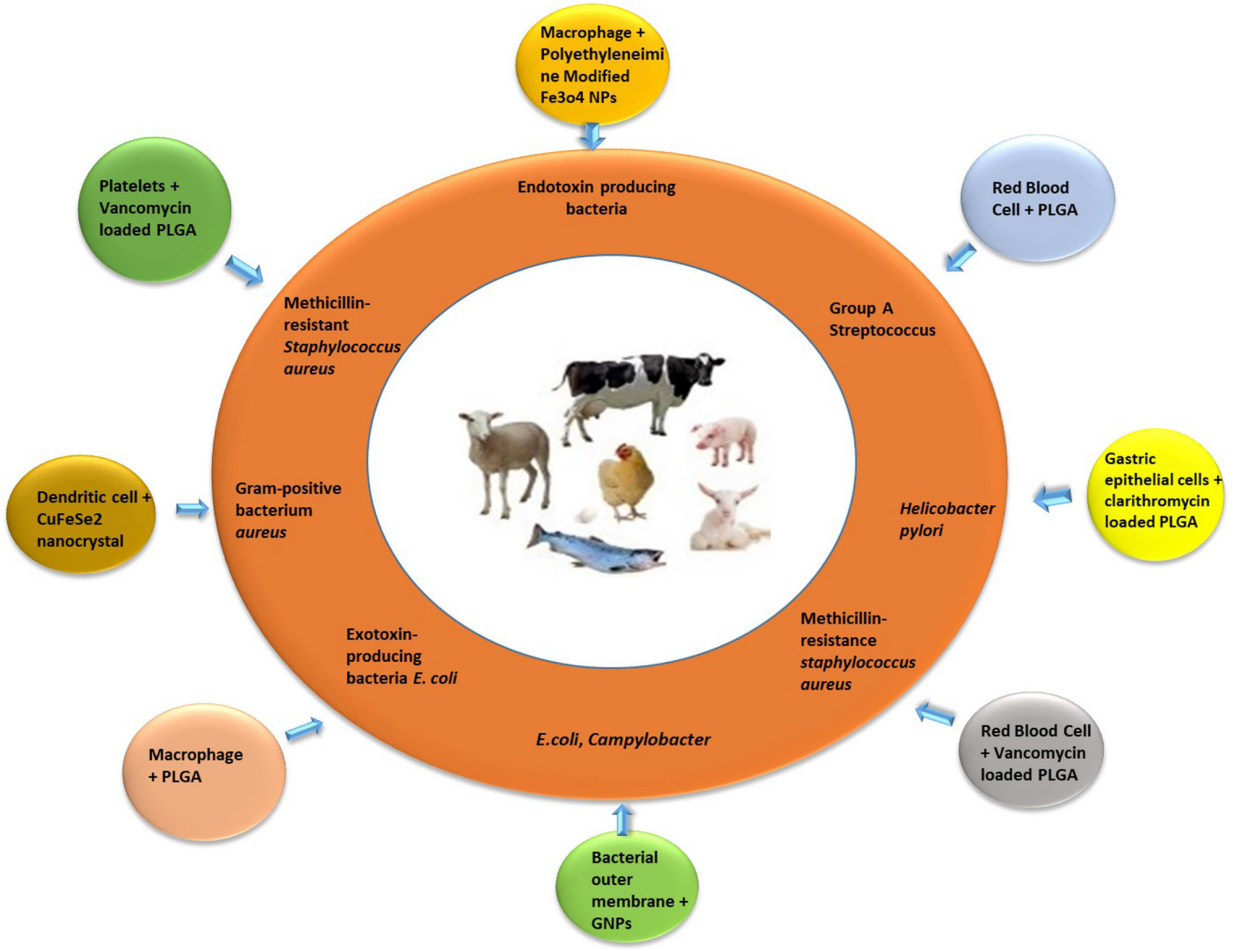
Bioinspired and biomimetic nanotherapies against specific bacterial pathogens of food animals.

Biomimetic manufacturing of nanoparticles is a successful and rapidly developing field of nanotechnology (58). Depicting the concept of specific attachment sites and translocation protocols by pathogenic bacteria and host mammalian cells, the biomimetic nanoparticles showed several pharmacological functions like improved accumulation at site of infection/inflammation (78), efficient drug delivery to the target (79, 80), prolonged duration of circulation (81), and eliminate off-target adverse effects in the healthy host tissues (82). Thus, resulting in development of more effective and targeted nanoplatforms. All over the world, researchers have been working on bioengineered coated nanoparticles with special focus on antibacterial therapy (83, 84). The effectiveness, long-term safety, biopharmaceutical, and pharmacokinetic profiles of newly developed biomimetic nanotherapies are being confirmed by comprehensive anti-bacterial studies (85) (Table 1, Figure 1).

**Table 1:** Antibacterial cell membrane-coated nanoparticles against the food borne pathogens.

**Food borne pathogens**	**Bioinspired therapeutic approach**			**Disease(s) of food animals**	**Host reservoir**	**References**
	**Cell membrane**	**Nano-core**	**Cargo**			
Methicillin resistant *Staphylococcus aureus*	Platelet	PLGA	Docetaxel and Vancomycin	Soft tissue infection and mastitis	Poultry, pig, cow, sheep, goat, buffalo, sea food, human; NA	(54)
	RBC	PLGA	–	Sepsis, soft tissue infection and mastitis		(86)
	RBC	Fe_3_O_4_	–	Mastitis, septicemia arthritis, localized abscess formation		(40)
	Cholestrol enriched RBC	Cholestrol	Vancomycin	Skin infections, Mastitis		(87)
*Campylobacter jejuni*	*Campylobacter jejuni* membrane	PLGA	–	Gastrointestinal hemorrhage, pancreatitis cholecystitis, endocarditis, osteomyelitis meningitis	Chicken, swine, cattle, sea food, human	(88)
Group A Streptococcus	RBC	PLGA	–	Sepsis, pharyngitis, necrotizing fasciitis, impetigo and toxic shock syndrome	Fish, pig, cattle, human being	(89)
Group B Streptococcus	RBC	PLGA	–	Neonatal early-onset sepsis, intra mammary infection, respiratory infections, urinary tract infections, joint and bone infections, meningitis and bacteremia	Bovine and human	(90, 91)
*Helicobacter pylori*	Gastric epithelial cell membrane	PLGA	Clarithromycin	Gastric ulcer, lymphoma and gastric cancer in humans, Commensal in Sheep, cow pigs and camel sometimes cause digestive infection	Sheep, cow, pigs and camel milk, human	(92)
Endotoxin (LPS) producing bacteria including strains of *Escherichia coli, Staphylococcus*	Macrophage	Ca_3_(PO_4_)_2_-Fe_3_O_4_@TiO_2_	–	Osteomyelitis, sepsis, mastitis	Poultry, goat, sheep, broiler, pig, human	(39, 93)
	Endothelial cells	PLGA	Dexamethasone	Sepsis		(94)
	Macrophage	Fe_3_O_4_	–	Endotoxemia, sepsis, shock, and multiple organ dysfunction		(95)
	Macrophage	PLGA	–	Sepsis		(48)
*Staphylococcus aureus*	Macrophage	Gold-silver nanocage	–	Mastitis, septicemia arthritis, localized abscess formation	Cattle, poultry, chicken, sheep, goat, pigs, and humans	(96)
	*Staphylococcus aureus*	PEG	Rifampicin	Mastitis, septicemia, septic arthritis		(97)
		PLGA	Vancomycin			
	*Staphylococcus aureus* treated dendritic cells	CuFeSe2	–	Staphylococcus induced osteomyelitis		(98)
*Pseudomonas aeruginosa*	Macrophage	Proteins secretions of Pseudomonas aeruginosa	–	Otitis and urinary tract infections, mastitis	Dairy cows, dogs; human	(99, 100)
*Klebsiella pneumoniae*	RBC	PLGA	Ciprofloxacin	Sepsis, urinary tract and pneumonic infections.	Chicken, pork, turkey and human	(51)
*Salmonella Enteritidis*	*Salmonella enteritidis* outer membrane protein	Chitosan	–	vomiting, high fever, diarrhea, and death in immunocompromised human patients	Poultry products (meat, eggs), layer, broiler chicken, and human	(49)
	*Salmonella enteritidis* outer membrane protein and flagellin protein (oral formulation)	Chitosan	–	Nausea, high temperature, diarrhea, vomiting and death in humans		(101)

PLGA, poly(lactic-co-glycolic acid); RBC, red blood cell; CuFeSe_2_, Copper iron sulfide; PEG, polyethylene glycol; Ca_3_(PO_4_)_2_, Calcium phosphate; Fe_3_O_4_, Iron (II, III) oxide; TiO_2_, Titanium dioxide.

## Major food animal pathogens treated through cell membrane-coated nanoparticles

### Klebsiella pneumoniae

*Klebsiella pneumoniae* is commonly considered a responsible pathogen for nosocomial and community-acquired infections, including liver abscesses, infections of urinary tract and lower respiratory tract (102). The causative bacteria may be found in several niches like soil, meat, feaces, skin and intestine of livestock, and sea food animals (103). Antimicrobial-resistant species of *K. pneumoniae* have been found in various food products, including raw meat (bovine, chicken, and pork), sea food, vegetables, and ready-to-eat meals (104–108). World Health Organization (WHO) has listed cephalosporins and carbapenems resistant *K. pneumonia*e strain, as one of the top threats to public health globally. Thus emphasizing the urgent need for the discovery and development of new therapeutic agents (109, 110). Sepsis is a systemic inflammatory response syndrome caused by infection, with high incidence and mortality. Therefore, it is necessary to carry out an effective anti-infection treatment. In a recent research work, the scientists designed and developed a new biomimetic nanomedicine and named it γ3-RBCNPs. This bioinspired antibacterial agent involves the loading of ciprofloxacin into PLGA nanoparticles and then wrapping with RBCM. These functionalized nanoformulations specifically target intercellular adhesion molecule-1 (ICAM-1) *via* γ3 peptide on their surface and ultimately eradicate the *K. pneumoniae* with the help of loaded ciprofloxacin. The RBC membrane coating over the nanoparticles offers the properties of prolonged circulation time and immune escape (45, 46). Further investigations indicated that γ3-RBCNPs have good antibacterial efficacy, good bio-safety, and long half-life in mice mode (51).

### Salmonella enteritidis

It is a foodborne pathogen, possess several zoonotic serotypes as well as a wide range of host organisms including poultry, ovine, bovine, and humans (111). It is the most common type of infectious agent, causing animal and human Salmonellosis (112, 113). *S. enteritidis* is responsible for a reduction in egg production in poultry, leading economic losses to poultry industry (114, 115). Notably, *S. enteritidis*-infected meat and contaminated eggs are the primary cause of human Salmonellosis, so the control and treatment of Salmonellosis in the food animals would definitely lead to decline in cases of human Salmonellosis (116, 117). Moreover, the multi-drug resistant (MDR) strains of salmonella are emerging and their prevalence is also increasing day by day (118–120). In Egypt, the percentage of prevalence of multi drug resistant *S. enterica* was found to be 69.8% in the marketed meat samples in 2010, and 82.4 to 100% in 2020 (121–123).

Several therapeutic and preventive strategies have been considered to control Salmonella shedding in poultry and other livestock animals (124). It is expected that novel non-antibiotic therapeutic agents may reduce burden of bacterial colonization, environmental contamination and public health risk (125). Currently, Salmonella vaccines have been developed by coating of bacterial cell membranes over the chitosan nanoparticles. Scientists utilized *S. enterica* outer membrane proteins, flagellin proteins, and chitosan nanoparticles for the development of nano-vaccine against Salmonella (CNP-vaccine). Two experiments were performed, Experiment I was done to evaluate the optimal dose of CNP-vaccine to get a protective response against bacterial infection of *S. Enteritidis*. Whereas the Experiment II was performed to investigate the cross protection of nanoformulations against bacterial infection of *S. Heidelberg*. The results indicated that CNP-Salmonella vaccine was capable to raise the level of IgA and IgG in *S. Enteritidis* or *S. Heidelberg* infected broilers, thus showed cross-protection against both serovars of *S. enterica* (49).

## Methicillin-resistant *Staphylococcus aureus* (MRSA)

This is a highly dangerous drug-resistant pathogen which is responsible for a wide range of infections such as endocarditis, sepsis and bacteremia leading to high rate of mortality (126–128). In food animals MRSA was first detected in the early 1970s in Belgium and induced bovine mastitis (129). After that, several cases have been reported on MRSA infection and colonization in other food-chain animals such as poultry, cattle, sheep, goat and pigs, intimating MRSA as an imperative veterinary and zoonotic pathogen (130–132).

Molecular typing of *S. aureus* indicated that certain animal lineages are specific to host while some are able to colonize or infect a wide variety of animals (133). The most notable case is ST398, which was primarily detected among pigs, and afterwards was found in various food-chain animals as well as in humans. The isolates stated to date and carrying mecC mainly belonged to lineages in cattle. However, mec C has also been indicated in other food animals such as sheep and rabbits as well as among various companion animals (cats, guinea pigs, horses, and dogs) (134, 135).

The pathogenesis of *S. aureus* pathogenesis is fueled by lethal secretion of toxins including α-toxin, which damages the membrane by forming pores and targets epithelium and endothelium of leukocytes and platelets (136). Most antibiotics are ineffective in treating MRSA infections as MRSA has a good survival strategy and self-preservation (137, 138). Nano-drug delivery systems have emerged as a new method to overcome this barrier (135). Recently, a study was conducted to synthesize an innovative nano-drug delivery system against MRSA infection. The researchers developed silver metal-organic frame structure (Ag-MOF) of methylimidazole and silver nitrate. Then the framework was loaded with Vancomycin (Vanc) to form Ag-MOF-Vanc complex and finally coated with platelet vesicles to get platelet membrane covered nanoparticles PLT@Ag-MOF-Vanc. The prepared nanoformulations killed MRSA through various strategies, such as interfering with bacterial metabolism, inhibiting production of reactive oxygen species, destructing the structure of cell membrane, and inhibiting formation of biofilm. The coating of the platelet membrane buttoned up to the surface of the pathogenic bacteria (MRSA) and the sites of MRSA infection. The results indicated good therapeutic effect in the mouse MRSA pneumonia model, and showed no toxic effect (139).

A biomimetic nanodecoy strategy was developed to capture and neutralize *S. aureus* toxins. The Platelets membranes were isolated and coated over PLGA nanoparticles and the resulting formulation of nanospongee inhibit the platelet and macrophage damage, induced by toxins of *S. aureus*, thereby supporting activation of platelets, nitric oxide production, macrophage oxidative burst, and bactericidal activity. Moreover, nanosponge also helped in release of neutrophils which are trapped in the extracellular fluid by a pathogenic organism. Thus, the prepared platelet membrane coated nanoparticles (PNPs) provided the therapeutic benefits of cytoprotection and enhanced host resistance to *S. aureus* infection (50).

The cell membrane-coated nanoparticles have great potential for treatment of bacterial infections. However, infection of bones involved inflammation and loss of bone mass, so the treatment must have anti-inflammatory and osteoconductive agents (39). The membrane coating through ultrasonication and extrusion strategies reduced the functionality of plasma membrane, so for coating the nanoparticles, another protocol was introduced in 2021, which involved electroporation procedure to help in retaining the functionality of cell membranes. A composite of nanoparticles with bactericidal TiO_2_ and osteoconductive Ca_3_(PO_4_)_2_ properties was assembled and coated by macrophage membrane. The resulting macrophage membrane coated nanoparticles possess better functional membranous coating and showed significant bactericidal and anti-inflammatory potential against methicillin resistant *S. aureus* infection (39).

### Escherichia coli

*Escherichia coli* (*E. coli*) is both commensal and pathogenic type of bacteria existing in humans and animals (140). Shiga-toxin producing *E. coli* (STEC), known as *E. coli* 0157 is responsible for causing resistant infectious disease (141–144). The symptoms of disease include fever, nausea, cramps, vomiting, bloody or watery diarrhea and sometimes kidney failure leading to death. Cows especially calves, sheep, goats, pig and deer may transfer the infection to humans. It is a food-borne pathogen and people got infection by either having contaminated food, including unpasteurized milk and undercooked beef or by having direct contact with *E. coli* O157 from stool of calves and cattle (145, 146). Animals carry *E. coli* O157 in the intestine and then shed in stool but still appear healthy and clean. The germs can quickly contaminate the animals' skin, fur, feathers, and the areas where they live and roam. Animals can lead healthy life but may potentially spread *E. coli* O157 infection to other animals and humans. *E. coli* may cause bovine mastitis in cattle (147, 148) and infection can be graded from being a subclinical illness of bovine mammary gland to a serious systemic infection. The age and lactation stage of the cows are the major factors to be considered for determining the severity of coliform mastitis.

The currently available antimicrobial agents are not effective enough for treatment of *E. coli* mastitis. However, cephalosporins and fluoroquinolones have shown partial therapeutic benefits for treatment of *E. coli* mastitis (149). The use of both drugs is restricted to a limit in food animals with special instructions (150). In *E. coli* mastitis, a potential therapeutic agent is needed, scientists are working to design and develop biomimetic nanoformulations for the successful treatment of the *E. coli* instigated disease. The disguised nanoparticles were prepared by using macrophage membranes to specifically neutralize and deactivate lipopolysaccharides secreted by *E. coli*, pathogenic bacteria. The *in vivo* experimentation in endotoxemia-induced mice model demonstrated that the coated nanoparticles reduced immune response and the inflammatory reaction. In addition, the nanoformulation improved the survival rate. These macrophage membranes coated nanoparticles are broadly considered for the treatment of LPS associated infectious diseases (95).

### Helicobacter pylori

*Helicobacter pylori* is a spiral shaped gram negative bacterium which mainly colonize in the gastric environment (151). This highly virulent bacteria specie is responsible to cause ulcer, lymphoma and gastric cancer (152). The transmission route of the bacteria is still not known. However, studies were conducted in the past to evaluate the probability of zoonotic transmission. In a study, scientists isolated *H. pylori* from pigs, sheep, cow, and camel milk (153, 154) and the results showed that *H. pylori* may opt these animals as reservoirs. In another study, the prevalence and virulence of *H. pylori* was determined in the stomach of sheep, cow and goat. The zoonotic transmission of *H. pylori* was also examined. It was demonstrated in the results that cows, sheep, and human beings samples were found *H. pylori* positive; however the bacterium was not detected in goat samples. Moreover, the virulent *H. pylori* genotype (vacA s1a/m1a) linked to epithelial cell injury, was the predominant *H. pylori* genotype in cow, and goat population (155). *H. pylori* infection is the major cause of gastric and peptic ulcer, gastritis and gastric cancer, thus it is highly essential to treat the infection of food animals and humans with potential therapeutic agents. Currently, clarithromycin, amoxicillin and metronidazole are recommended for the treatment. However, mutations in *H. pylori* genes have led to the treatment failure (156).

Recently, a novel targeted nanotherapeutic agent was developed, inspired by the idea of natural interaction and adhesion of host and the bacteria. The plasma membranes of gastric epithelial cells are coated over chlarithromycin-loaded polymeric nanoparticles, the resulting biomimetic nanoparticles have the same surface properties as the epithelial cells and thus adhere to *H. pylori* bacteria. Thus, the nanoformulations provided the targeted drug delivery platform against colonized pathogenic bacteria. Thus, the bioinspired nanotherapeutic approach strategy reported here represented a novel drug delivery system to treat infectious disease of *H. pylori* (92).

Another versatile targeted therapeutic agent was synthesized by Zhang and his coworkers (157). The bioinspired moieties interfere with adhesion of bacteria to the host and thus evade the decorum of bacterial growth inhibition or killing, which may reduce the resistance development. The polymeric cores were wrapped with bacterial outer membranes resulting in synthesis of *H-pylori*-mimicking nanoparticles which compete with source bacteria for binding to gastric epithelial cells. Treatment of *H-pylori*-mimicking nanoparticles with gastric epithelial cells enhance the target adhesion and the efficacy is dependent on coated nanoparticles concentration and dosing (157).

### Campylobacter jejuni

*Campylobacter jejuni* is highly responsible for foodborne zoonosis and is associated with handling and consumption of the poultry meat. The bacteria colonizes in the intestine of chicken and thus slaughtering process resulted in fecal contamination of the carcasses (158). One strategy to control *C. jejuni* infection is to halt the bacterial colonization of broilers (159).

The extent of contamination is directly linked to the number of bacteria in the cecal content of broiler chicken and the number of bacteria on the carcasses and cut meat pieces (160). Therefore, Campylobacter load is reduced at poultry production level before slaughtering (161). Several antibiotic drugs have been considered for the treatment and control of the infection but still the infection prevails due to the existence of resistant strains of bacteria.

Several therapeutic and preventive strategies have been developed against *C. jejuni* colonization in poultry (159, 160, 162–164). In a study, biodegradable and biocompatible poly (lactide-co-glycolide) (PLGA) compound was used to prepare nanoparticle and then the synthesized nanoparticles were wrapped with outer membrane proteins of *C. jejuni*. The prepared coated nanoparticles were then administered through oral/subcutaneous routes. The results were interpreted based on intestinal colonization of *C. jejuni* in chicken. The *C. jejuni* colonization in cecal and cloacal contents was reduced post administration of prepared nano vaccine. It was concluded that nanoparticles encapsulated with outer membrane proteins of *C. jejuni* may be considered as a potential candidate vaccine for controlling the colonization of *C. jejuni* in chickens (88).

Considering the dynamic interaction of pathogen with host as well as understanding the versatile functionality of bacterial outer membrane proteins for intestinal adherence and invasion, bacterial outer-membrane vesicles have become a potential candidate for vaccine targeting against *C. jejuni* (165). The chitosan-nanoparticles were coated with outer-membrane vesicles and then evaluated in mouse model for induction of specific immune response against *C. jejuni*. The results indicated that intragastric delivery of chitosan-coated outer-membrane vesicles impart significant immune protection (166).

Further, immunization with the outer membrane vesicles resulted in potent cellular responses with an increased CD4+ and CD8+ T cell population. Moreover, significant upregulation of IFN-γ and IL-6 gene expression suggests that mucosal delivery of outer membrane vesicles promotes a Th1/Th2 mixed-type immune response. Together, as an acellular and nonreplicating canonical end product of bacterial secretion, mucosal delivery of outer membrane vesicles may represent a promising platform for developing an effective vaccine against *C. jejuni* (166).

## Group A streptococcus and Group B streptococcus

The strains of Streptococcus cause mild to severe bacterial disease in animals as well as in human beings. The pathogenic organism is not host-specific as some strains which are associated with animals can be frequently found in humans (167, 168). Streptococcus infection may rapidly develop into a lethal disease despite administration of commercially available drugs (antibiotics and palliative medication). The streptococcus bacteria possess detrimental virulence factors, such as Group A streptococcus pore-forming streptolysin O and Group B streptococcus pore-forming toxin β-hemolysin/cytolysin. The toxins adversely effects the host tissues including failing of blood brain barrier, injury of lung cells, and apoptosis of immune cells (90). Many zoonotic cases of streptococci are sporadic, but some fish-linked strain of *S. agalactiae* are involved in outbreaks (169, 170). Pig associated strain of *S. suis*, has emerged as a major environmental pathogen causing deadly shock-like syndrome, septicemia, streptoccoccal meningitis, and other human diseases after contact with infected animals or derived food products, especially in the Asian countries (171–174). Moreover, in case of cattle, streptococci are declared to be the virulent bacteria causing bovine mastitis throughout the world (175, 176).

Several classes of antibiotic drugs are considered for the treatment of infected animals. First-line treatment for streptococci infection is penicillins either alone or in combo with tetracyclines, aminoglycosides, lincosamides, macrolides and fluoroquinolones (177). However, antibiotic-resistant phenotypes of streptococci have been reported in infected animals all over the world (178). New treatment strategies are being considered against streptococcal infection (179, 180), including a titratable detoxification therapy by a wraping of polymeric cores with donor red blood cell membranes to create biomimetic “nanosponges”. The polymeric cores of biomimetic nanosponge retain the same repertoire of receptors over the cell membrane and proposed a non-specific toxin decoy stratagem to neutralize and sequester several bacterial toxins and pro-inflammatory chemokines of the host. The prepared nanosponge was evaluated to neutralize and sequester streptolysin O toxin of Group A Streptococcus (GAS) and inhibit the functioning of later virulent bacteria. Moreover, this therapeutic intervention inhibited toxin-induced apoptosis of macrophage and enhanced neutrophil trapping process leading to enhanced killing of pathogenic bacteria by phagocytic cells. *In vivo* studies of biomimetic nanosponge showed that the local administration of the prepared nanosponge reduced lesion size and bacterial colony-forming unit in the GAS-infected mouse model. Thus, the application of a toxin decoy assisted in the inactivation of secreted toxin and presented a novel approach to directly target virulence in severe GAS infections (89).

In the recent research work, red blood cell membrane vesicles were isolated and coated over polymeric nanoparticles to formulate biomimetic nanosponge. The prepared nanosponges proved to be successfully alleviate a sequence of toxic events like the hemolytic activity of living Goup B streptococcus bacteria, injury of lung epithelial and production of streptococcus -induced macrophage IL-1β. The results also indicated that red blood cell membrane-coated nanoparticles might be considered a first-in-class treatment option for streptococcal bacterial infection (90).

## Conclusion

Traditional anti-infection therapeutic strategies mainly involved administration of antibiotics. However, emergence of drug-resistant bacteria and increasing endemic of lethal bacterial infections raised the importance of development of novel therapeutic agents for infectious diseases. Recent advances in nanotechnology, resulted in synthesis of a variety of nanoscale cell-membrane bagged nanoparticles. Several efforts have been directed toward nanoparticle functionalization by successful protection during the interaction of bioinspired nanoparticles with pathogens or exo/endotoxin. Food-borne zoonotic pathogens have been targeted by biomimetic nanoparticles and showed potential antibacterial activity.

## Author contributions

SA contributed to the design/plan of the work. KA and SA collected and interpreted the data and drafted, revised, and approved the final version of the article to be published.
